# Chlorhexidine Resistance or Cross-Resistance, That Is the Question

**DOI:** 10.3390/antibiotics12050798

**Published:** 2023-04-22

**Authors:** Hadeel Mohammed Abbood, Karolin Hijazi, Ian M. Gould

**Affiliations:** 1 Institute of Dentistry, School of Medicine, Medical Science and Nutrition, University of Aberdeen, Aberdeen AB25 2ZR, UK; 2 College of Dentistry, Tikrit University, Tikrit 34001, Iraq; 3 Department of Medical Microbiology, Aberdeen Royal Infirmary, Aberdeen AB25 2ZN, UK

**Keywords:** chlorhexidine, biocides (disinfectants), antibiotic resistance, cross-resistance

## Abstract

Chlorohexidine (CHX) is a widely used biocide in clinical and household settings. Studies over the last few decades have reported CHX resistance in different bacterial species, but at concentrations well below those used in the clinical setting. Synthesis of these findings is hampered by the inconsistent compliance with standard laboratory procedures for biocide susceptibility testing. Meanwhile, studies of in vitro CHX-adapted bacteria have reported cross-resistance between CHX and other antimicrobials. This could be related to common resistance mechanisms of CHX and other antimicrobials and/or the selective pressure driven by the intensive use of CHX. Importantly, CHX resistance and cross-resistance to antimicrobials should be investigated in clinical as well as environmental isolates to further our understanding of the role of CHX in selection of multidrug resistance. Whilst clinical studies to support the hypothesis of CHX cross-resistance with antibiotics are currently lacking, we recommend raising the awareness of healthcare providers in a range of clinical disciplines regarding the potential adverse impact of the unfettered use of CHX on tackling antimicrobial resistance.

## 1. Introduction

Chlorhexidine (CHX) is a chemical agent composed of two biguanide compounds linked by a hexamethylene bridge. This structure confers a positive charge with basic properties on both sides [1]. CHX has three main forms: digluconate, acetate, and hydrochloride, which all are water soluble [2]. It was introduced as a medical antiseptic in the UK in the early 1950s and used for the first time to inhibit the formation of dental plaque in 1969 [3,4]. Currently, CHX is widely used for pre-operative skin disinfection, decontamination of skin and mucous membranes in intensive care units, impregnation of medical devices such as catheters, and disinfection of inanimate surfaces [5]. In addition, CHX is used in dentistry in the treatment of periodontal diseases as a mouthwash and toothpaste as well as in gels, varnishes, and sprays [2].

Over the last few decades, there have been numerous reports suggesting an increase in bacterial resistance against CHX in a variety of species [5,6]. However, no definitive findings informing universal clinical practice have emerged. The aim of this review was to gather the most up-to-date information regarding CHX resistance and more broadly the effect of CHX on the emergence and spread of antimicrobial resistance.

## 2. Mechanism of Action

CHX is lipophilic and positively charged. These two properties enable the interaction of CHX with negatively charged phospholipids and lipopolysaccharides (LPS) of the bacterial cell wall or the outer membrane [7]. At low concentrations (0.2%), this interaction leads to cell wall damage and leakage of low molecular weight constituents, such as potassium and phosphate. At higher concentrations (2% and higher), CHX enters the plasma membrane causing cytolysis, release of intracellular components, and coagulation and precipitation of the cytoplasmic proteins due to the formation of phosphate compounds, which in turn leads to cell death [8].

## 3. Spectrum of Action

CHX is a broad-spectrum biocide and is active against bacteria, fungi, and some protozoa. CHX is most effective against Gram +ve bacteria and, to a lesser degree, against Gram −ve bacteria and fungi [5]. This is due to the presence of the outer membrane in Gram −ve bacteria [9] and the complexity of the cell wall in fungi that limits intracellular perfusion of CHX. Interestingly, fungi can be inhibited by low concentrations of CHX (25 µg/mL) [10,11,12] but are less susceptible at higher concentrations (1000 µg/mL) [12]. On the other hand, CHX showed no bactericidal activity against mycobacteria, even after exposure for longer than 120 min [13].

CHX has shown good antiviral activity against lipid-enveloped viruses, such as Herpes Simplex Virus (HSV), Human Immunodeficiency Virus (HIV), and Cytomegalovirus [14], but not non-enveloped viruses, such as Rotavirus and Poliovirus [15]. However, findings regarding the effect of CHX on SARS-CoV-2 are conflicting [16,17,18]. Earlier reports suggested lower CHX virucidal activity against SARS-CoV-2 on inanimate surfaces compared to other coronaviruses [17]. A clinical study showed a transient suppression of SARS-CoV-2 salivary viral load to a non-detectable level for 2 h in two patients after the use of a 0.12% CHX mouthwash, with viral loads reverting to higher levels within 4 h. [18]. The inhibitory effect of CHX mouthwash on salivary viral load in COVID-19 patients was supported by another report. However, the comparison of CHX mouthwash with a control mouthwash (0.9% NaCl) suggested that the viral load reduction observed in this study may be caused primarily by the mechanical action of the mouthwash rather than the antiviral impact of CHX [19]. Due to the small sample size in the existing trials, more studies are still needed to prove the effectiveness of CHX on SARS-CoV-2.

## 4. Mechanisms of Resistance to Biocides

Broadly, bacterial tolerance to biocides depends on the nature of the biocide itself and the target species. In addition, environmental characteristics, such as temperature and pH as well as biocide concentration and time of exposure, can have a considerable effect on bacterial tolerance [20]. For example, dental plaque bacteria showed decreasing viability with increasing concentration and time of CHX exposure [21]. Biocides are a diverse group of chemical substances that act through a variety of mechanisms, including lysis and the suppression of enzymatic activity. Biocides can target the cell wall, the cell membrane, and the cytoplasm. Some biocides, such as biguanides, including CHX, can act on more than one target based on the concentration of the biocide [22].

Structural resistance to antimicrobial agents, including biocides, can be controlled by intrinsic and extrinsic mechanisms:

Bacterial spore formation is one example of intrinsic antimicrobial resistance mechanisms potentially relevant to biocides; the presence of the cortex and the inner and outer coats of bacterial spores limit the penetration of many biocides [23]. However, some biocides can be sporicidal if used in high concentrations with long periods of contact. Quaternary ammonium compounds and bisbiguanides, including CHX, are considered sporistatic but not sporicidal at a high concentration, unlike formaldehyde, chlorine, and hydrogen peroxides [23,24]. The composition of the microbial cell wall or the presence of the outer membrane are other examples of intrinsic antimicrobial resistance. In mycobacteria, the presence of free lipids and a waxy cell envelope may be related to the reduced susceptibility to several biocides [25]. CHX is considered tuberculostatic even at high concentrations, whereas alcohols and formaldehydes are tuberculocidal [26]. Biofilm formation per se also contributes to an increased resistance to biocides due to adaptation to low-level nutrients and reduction of metabolic activity [27] and the layering structure hindering biocide penetration to deep layers [28].

Extrinsic mechanisms of biocide resistance are mediated by the acquisition of mobile genetic elements or mutations in chromosomal genes. Mobile genetic element acquisition is the most clinically relevant mechanism as it is thought to be driven by the selective pressure exerted by antimicrobial use in the community and hospitals. The types of phenotypic changes that arise from most, if not all, genetic changes are (i) the reduced uptake of the antimicrobial agent, (ii) the reduced permeability of the outer membrane, and (iii) an increase in efflux pump activity, which is described further in the following section [29].

## 5. Efflux Pump Activity

Efflux pumps are cell membrane-associated proteins which regulate substrate transport outside the bacterial cell and are a key mechanism of antimicrobial resistance. Efflux pump systems are naturally more complex in Gram −ve bacteria due to the presence of the outer LPS membrane and are therefore organized in tripartite channel-forming complexes [30]. Genes encoding efflux pumps are either borne on mobile genetic elements or chromosomally expressed. In the latter scenario, mutations in genes encoding efflux pumps can lead to overexpression, in turn resulting in decreased susceptibility to antimicrobial agents [29]. The cumulative effect of mutations over time, fundamentally induced by selective pressure of exposure to antimicrobials, drives high-level resistance detected in diagnostic antimicrobial susceptibility assays [31]. On the other hand, environmental pressure can induce transient resistance through the overexpression of efflux pumps, leading to bacterial persistence. This, in turn, leads to a higher frequency of spontaneous mutations and permanent resistance [32]. Five major families of efflux pumps have been described: (i) ATP-binding cassette (ABC) transporters are made up of two substrate-binding transmembrane domains and two nucleotide-binding domains that use ATP hydrolysis as source of energy [33]. LmrA was one of the first bacterial ABC transporters ever characterised. The overexpression of LmrA in *Escherichia coli* showed a reduced susceptibility to a broad range of non-related substrates, including ethidium and daunomycin [34]. MacAB-TolC is another example of an ABC transporter. In *E. coli*, the expression of MacAB-TolC increases resistance to macrolides [35]. With regards to the relevance of this efflux pump family to biocide resistance, the P-type ATPase SilP efflux protein has been associated with reduced susceptibility to silver [36]. (ii) The small multidrug (SMR) resistance family is a group of small homologous proteins that shows specificity to lipophilic compounds such as quaternary ammonium compounds (QAC) [37]. These proteins allow a variety of compounds to efflux through the proton motive force or electrochemical gradient [38]. QacC is an SMR transporter encoded by a plasmid-borne gene in *Staphylococcus.* This transporter has been associated with a reduced susceptibility to a range of biocides, including benzalkonium chloride, but not CHX [38,39]. (iii) The major facilitator superfamily (MFS) is the largest family of secondary transporter proteins. It is a group of single polypeptide proteins that can transport a range of solutes by the proton motive force [40]. The MFS family includes EmrAB-TolC and QacA/B, the latter of which can mediate the transport of biocides such as benzalkonium chloride and CHX [38,39]. (iv) The resistance nodulation cell division (RND) family is a group of active efflux pumps responsible for drug resistance in Gram −ve bacteria [41]. AcrAB-TolC is an RND transporter tripartite consisting of an inner-membrane protein AcrB that interacts with periplasmic protein AcrA and the outer membrane channel TolC to extrude β-lactam, tetracycline, and fluoroquinolone antibiotics [42]. Triclosan is one of the biocide substrates for the AcrAB-TolC system [43]. (v) The Multidrug and Toxin Extrusion (MATE) family is a group of transporter proteins that protects the cell from different types of antimicrobials [44]. PmpM is an exemplar MATE transporter, the binding activity of which extends to biocides such as benzalkonium chloride [45]. Resistance to antimicrobials can be augmented by the synergistic effect of different transporters. For example, the co-presence of *qacA* and *qacC* has been long shown to reduce susceptibility against benzalkonium chloride [39]. Table 1 enumerates examples of major efflux pump families in Gram −ve and Gram +ve bacteria and their relevance to biocide transport.

## 6. Mechanism of Resistance to CHX

Decreased susceptibilities to CHX in a range of clinically significant bacteria have been reported for the last couple of decades. However, there have been no definitive conclusions informing clinical practice at universal level. Three mechanisms of reduced susceptibility/resistance to CHX have been described: increased efflux pump activity, change in membrane permeability, and biofilm formation [46]. All three mechanisms are genetically controlled, at least in part; examples of genes associated with reduced susceptibility to CHX are listed below and in Table 2:

1. Increased efflux pump activity:
  i. Upregulation of RND efflux pumps. The mutation of genes encoding RND pumps, such as *marA* mutations, leads to the upregulation of RND protein pumps AcrAB-TolC through the overexpression of the MarA protein in *E. coli* [47]. This multidrug efflux pump system controls the efflux of antibiotics, oil solvents, and biocides, including CHX [48]. Clinical isolates of *Acinetobacter baumanii* showed a more than 10-fold increase in the CHX minimum inhibitory concentration (MIC) compared to susceptible isolates carrying RND efflux pump-encoding genes *adeB*, *adeJ*, and *qacE*. In the same study, inactivation of *adeB* and *adeJ* reduced the MIC by 8-fold and 2-fold, respectively [49]. These genes were found to play the same role in susceptibility to benzalkonium chloride, ethidium bromide, and acriflavine [50].
  ii. Acquisition of SMR pumps. The over-expression of these pumps, especially QacE, QacEΔ1, and EmrE efflux pumps, was seen in association with CHX MIC increase in *E. coli* biofilms when compared to planktonic and colony growth [50]. Deletion of *adeS* (encoding a putative SMR pump) in clinical isolates of *A. baumanii* showed a 2-fold increase in CHX susceptibility [51].
  iii. Acquisition of MFS pumps. QacA and QacB pumps are frequently identified in *Staphylococcus* isolates displaying reduced susceptibility to CHX. The genes encoding QacA and QacB are acquisitional plasmid-borne genes implicated in horizontal transfer between different species of *Staphylococcus* [52]. The genes that encode the two pumps are usually described as *qacA/B* in view of their high homology [53].
  iv. AceI pump. This recently discovered prototype of the Proteobacterial Chlorhexidine Efflux (PCE) family showed specificity to CHX amongst other substrates. In *E. coli*, *aceI* overexpression was associated with a reduced susceptibility to CHX [46].
2. Change in membrane permeability [54]:
  i. Change in porin profile. Porins are channels for substrate transport formed by outer membrane proteins (OMP) [54]. Some porins can also play an important role in outer membrane integrity by interacting with peptidoglycans such as OmpA [55]. In *Pseudomonas stutzeri*, changes in OMP profile were associated with increased CHX MIC [54]. *E. coli* gradually adapted in CHX-containing culture medium showed >2-fold upregulation in *ompX* and *ompA* and downregulation of *ompF* and *ompT* compared to the non-CHX-adapted strain [56]. OmpF is one of the non-specific porins in the outer membrane that form a complex with MlaA and allow the uptake of hydrophilic substrates, such as β-lactam antibiotics and CHX [56,57].
  ii. Loss of MlaA. MlaA in *E. coli* binds to OmpC/F to form the Mla intermembrane phospholipid transport system. The main function of this complex is to maintain asymmetry of the outer membrane lipids in Gram −ve bacteria by retrograde transport of phospholipids from the outer membrane and retention of LPS [57,58]. Inactivation of this retrograde transport channel resulted in reduced susceptibility to CHX, thereby implicating it in CHX cellular uptake. [56,59].
3. Bacterial biofilm formation [60]:
  i. Extracellular DNA (eDNA) is an important component of the biofilm. Its negative charge promotes non-specific binding to cationic antimicrobials, including CHX, which prevents CHX from reaching their target microorganism [61].
  ii. Biofilm formation promotes the upregulation of MDR efflux pumps [6], which bind a broad spectrum of antimicrobial agents. As outlined in the later section, the breadth of MDR substrate specificity is thought to underpin cross-resistance to biocides and antibiotics.
  iii. The high abundance of extracellular polysaccharides forms a mechanical obstacle to the penetration of CHX into deep layers of established bacterial biofilms [62]. Antimicrobial diffusion into thick layers of biofilm may be delayed, thus exposing bacteria to sub-bactericidal concentrations that give rise to spontaneous mutations, causing antimicrobial resistance [63]. These conditions can also induce the expression of antimicrobial deactivating enzymes in the polysaccharide matrix [64,65].

**Table 2:** Genes associated with reduced susceptibility to CHX detected in different bacterial species.

	*Enterococcus* spp.	*E. coli*	*Salmonella* spp.	*Pseudomonas aeruginosa*	*Acinetobacter baumanii*	*Staphylococci*	*K. pneumonia*
*qacA/B*	[66]					[52,67]	
*qacG*				[68]		[69]	
*qacH*						[70]	
*qacE*, *qacED1*	[66]	[50]		[68]	[52]		
*adeB, adeJ*					[49,52]		
*cepA*				[68]			
*aceI*		[49]			[49,71]		
*acrA*, *acrB, tolC*			[43]				[72]
*mlaA*		[59]					
*ompF, ompC*		[59]					
*sigV*	[66]						
*gasp65*	[66]						
*emeA*	[66,73]						
*mdeA*						[74]	
*mepA*						[74]	
*fabV*				[75]			
*fabI*					[71]		
*abeS*					[54]		
*abeM*					[71]		
*efrA/B*	[73]						
*ramA*							[72]
*pmrC*			[76]				

## 7. CHX Versus Other Biocides

Few studies have examined and compared the susceptibility to different biocides in clinically relevant bacterial species, regardless of the different mechanism of action of these biocides. Formaldehyde, benzalkonium chloride, triclosan, and CHX were the most widely assessed in comparative studies of biocide antimicrobial activity, but the findings are conflicting. Several studies suggested that CHX has superior activity to other biocides. Tattawasart et al. compared cetylperidinium chloride (CPC) to CHX activity in *Pseudomonas* spp. and showed a lower MIC for CHX when compared to CPC [54]. A study in healthy volunteers showed the superior skin disinfection efficacy of CHX compared to triclosan and recommended the use of CHX as an antiseptic before surgical procedures [66]. Another study showed the lowest MIC_50/90_ for CHX compared to benzalkonium chloride, triclosan, and formaldehyde in both *Enterococcus faecalis* and *Enterococcus faecium* cultures. Epidemiological cut-off values of CHX were also lower for both species compared to other biocides and higher or equal than MIC values (Table 3) [77]. Importantly, this study included various isolates from healthy volunteers, patients, and environmental sources (sewages). Another study compared CHX to benzalkonium chloride and hydrogen peroxide in clinical vancomycin-resistant and susceptible isolates of *Enterococcus faecium* [75]. This study reported that vancomycin-resistant *Enterococci* (VRE) were less susceptible to CHX and benzalkonium chloride than vancomycin-susceptible strains (VSE). Morrissey et al. studied a variety of bacterial and fungal species from different sources and countries. The study reported higher MIC_90_ values for CHX compared to benzalkonium chloride and triclosan in *Salmonella* spp., *K. pneumonia*, *Enterobacter* spp., and *Enterococcus* spp. but not in *E. coli* and *S. aureus* for which the MIC values were lower than or equal to benzalkonium chloride but higher than triclosan [78]. The same study reported the highest CHX MIC in *E. coli*, *E faecalis*, and *K. pneumoniae* compared to the other species. This study provides insight on CHX susceptibility trends in different microbial species from different geographical and temporal settings; importantly, they tested physiological concentrations of biocides rather than in vitro sub-culture in increasing biocide concentrations. Although the study used different isolates from different time periods, there was no direct comparison of biocide susceptibility on temporal bases within the same species. A recent systematic review showed a decreased susceptibility to CHX overtime [5].

Regarding fungi, the systematic review by Buxser et al. showed no evidence of reduced susceptibility to CHX in *Candida albicans* over time (showing a slight increase in CHX susceptibility over the past 60 years) [5]. When the antifungal activity of CHX was compared with other biocides, CHX was less active against *C. tropicalis* and *C. krusei* when compared to CPC [79]. In another study, the MIC values of *C. albicans* cultures were higher for CHX compared to benzalkonium chloride but lower than triclosan [78]. These findings are consistent with the accepted knowledge that CHX is less active against fungi when compared to Gram +ve bacteria.

It is germane to highlight the regulatory status of triclosan, which has been used by many studies as a comparator to study the molecular mechanisms of reduced susceptibility of CHX. In response to several reports on the poor safety profile of triclosan as well as bacterial resistance [43,80,81], in addition to insufficient evidence on triclosan efficacy, a 2017 FDA ruling mandated that triclosan is excluded from over-the-counter (OTC) hygiene products [80]. Notwithstanding this ruling, triclosan is still contained in certain toothpastes, and the FDA has acknowledged the role of this agent in the prevention of gingivitis [82]. The use of triclosan in oral hygiene products should be reconsidered particularly in light of evidence that triclosan accumulates on nylon bristles of toothbrushes [83].

Table 3 summarises the antimicrobial activity of CHX vs. other biocides in key pathogens.

Comparative studies between biocides carry a lot of challenges as biocides are used topically and are naturally more vulnerable to external influences such as temperature, time of contact, and concentration. In addition, the testing of mixed microbial communities colonising biological as well as inanimate surfaces presents a further challenge due to the different levels of CHX susceptibility in different species driven by intrinsic and acquired mechanisms. These variables represent a major challenge in the interpretation of the biological significance of studies, particularly when comparing clinical and environmental isolates. The studies that have compared clinical and environmental isolates have shown clear differences in antimicrobial resistance patterns. A study of antimicrobial resistance time trends in environmental versus clinical isolates of different bacterial species showed an earlier occurrence of highly prevalent antimicrobial resistance genes compared to clinical isolates [84]. Others showed that clinical isolates of *P. aeruginosa* were more resistant to antimicrobial agents than environmental isolates and that resistance was associated with different genetic profiles [68]. Altogether, these studies draw attention to the importance of comparative studies of clinical and environmental isolates. With regards to the putative genetic determinants of susceptibility to multiple biocides, biocide tolerance-associated genes (BTA), which include *sigV*, *gsp65*, *emeA*, *qacA/b*, *qacD*, and *qacC*, have been associated with an increased tolerance to multiples biocides, including CHX [77,85]. As discussed in the following section, the broad-spectrum activity associated with genes such as the BTA group raises concerns regarding cross-resistance between biocides and antibiotics. Biocide exposure can induce the overexpression of BTA genes which due to their non-specific activity can result in cross-resistance [86].

**Table 3:** MIC and ECOFF of CHX and other biocides in different microorganisms.

Microorganism	Source of Isolates	Chlorhexidine			Benzalkonium Chloride			Cetylpyridinium Chloride			Formaldehyde			Triclosan			H_2_O_2_			Ref.
		MIC	MIC_90_	ECOFF	MIC	MIC_90_	ECOFF	MIC	MIC_90_	ECOFF	MIC	MIC_90_	ECOFF	MIC	MIC_90_	ECOFF	MIC	MIC_90_	ECOFF	
*Enterococcus Faecalis*	Clinical and environmental		8 µg/mL	8 µg/mL		16 µg/mL	16 µg/mL					256–512 µg/mL	512 µg/mL		16–32 µg/mL	32 µg/mL				[77]
	Clinical	64 g/L	32 g/L		8 g/L	4 g/L								16 g/L	8 g/L					[78]
*E. faecium*	Clinical and environmental		4–8 µg/mL	8 µg/mL		16 µg/mL	16 µg/mL					256–512 µg/mL	512 µg/mL		8–16 µg/mL	32 µg/mL				[77]
	Clinical	32 g/L	16 g/L		8 g/L	8 g/L								32 g/L	8 g/L					[78]
*Vancomycin susceptible Enterococci*	Clinical	≤4 mg/L			2–8 mg/L												45–65 mg/L			[75]
*Vancomycin Resistant Enterococci*	Clinical	≥4 mg/L			4–8 mg/L												40–64 mg/L			[75]
	Human wastewater	2 µg/mL			8 µg/mL							128 µg/mL			8 µg/mL					[87]
*C. tropicalis*	Clinical	75 µg/mL						66 µg/mL												[79]
*C. krusei*	Clinical	150 µg/mL						33 mg/mL												[79]
*C. albicans*	Clinical	16 g/L	8 g/L		16 g/L	4 g/L								16 g/L	8 g/L					[78]
*P. aeruginosa*	Clinical		64 µg/mL	64 µg/mL		1024 µg/mL	1024 µg/mL					512 µg/mL	512 µg/mL		512 µg/mL	512 µg/mL				[85]
	Clinical (includes resistant to CHX and resistant to cetylpyridinium chloride)	25 mg/L						500–1500 mg/L												[54]
*A. baumanni*	Clinical	8–128 µg/mL	64 µg/mL		4–32 µg/mL	32 µg/mL								2-> 256 µg/mL	128 µg/mL			47–376 µg/mL	94 µg/mL	[87]
*E. coli*	Clinical	64 g/L	16 g/L		64 g/L	32 g/L								2 g/L	0.5 g/L					[78]
*P. stuzeri*	Clinical (includes resistant to CHX and resistant to cetylpyridinium chloride)	2.5–100 mg/L						25–250 mg/L												[54]

## 8. Cross-Resistance to Other Antibiotics

With some exceptions, CHX is still unfetteredly used in hospitals and in oral health care on the backdrop of suggestions of cross-resistance between CHX and antibiotics [88,89,90].

An evaluation of antibacterial activity of CHX, benzalkonium chloride, and hydrogen peroxide in VRE versus VSE reported lower susceptibility to CHX and benzalkonium chloride and an increase in efflux pump activity in VRE [75]. Another study reported marginally lower CHX susceptibility in MRSA vs MSSA but showed no difference between VRE and VSE, notwithstanding that the source of isolates in this historical study was not clearly reported [73]. Of note, these two studies used different CHX salts (digluconate and diacetate, respectively). Further studies are required to investigate the longitudinal trend of VRE reduced susceptibility to CHX. Another study reported a co-occurring increase in CHX MIC and resistance to gentamicin associated with the presence of *efrA/B* in clinical isolates of *Enterococci* compared to faecal isolates collected from healthy volunteers [91].

**Figure 1:**
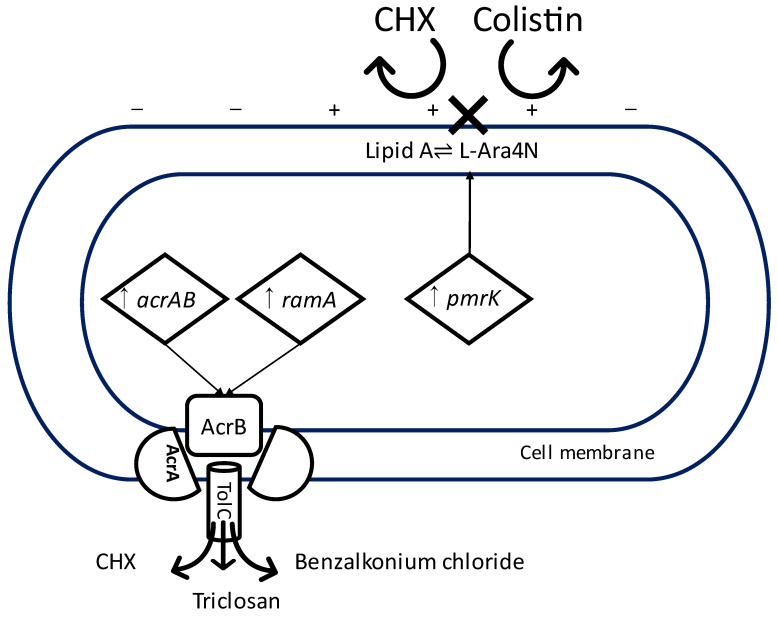
Diagram illustrating two possible mechanisms of cross-resistance between CHX and other antimicrobial agents in K. pneumonia. Upregulation of pmrK leading to the addition of L-Ara4N to lipid A and a change in the electrostatic charge of the cell membrane, repelling binding of positively charged chlorhexidine and colistin. Upregulation of acrAB and ramA leading to the increased activity of multidrug efflux pump AcrAB-TolC expelling chlorhexidine, triclosan, and benzalkonium chloride. CHX = chlorhexidine, ↑ = upregulation, X = failure of binding. −,+ = the electrostatic charge of the cell membrane.

With regards to *K. pneumoniae*, there have been reports of cross-resistance between CHX and colistin used for the treatment of carbapenem-resistant infections [89,92]. The mechanism of action of colistin is relatively similar to CHX in that it binds to the negatively charged LPS of the cell membrane, causing leakage of intracellular content [93]. Cross-resistance between CHX and colistin is thought to be driven by the upregulation of *pmrK*, which is associated with a decrease of LPS anionic charge by the addition of L-Ara4N to the phosphate of lipid A [76,94,95] (Figure 1).

Cross-resistance between CHX and antibiotics has also been reported in *Pseudomonas stutzeri* associated with eye infections secondary to the use of contaminated cosmetic materials. *P. stutzeri* is mostly highly sensitive to antibiotics and biocides [96]. However, gradual exposure of *P. stutzeri* to CHX in vitro resulted in sustained CHX resistance for more than 6 weeks [97]. In addition, cross-resistance to other biocides and antibiotics, such as benzalkonium chloride, triclosan, polymyxin, gentamicin, erythromycin, and ampicillin, was reported [97]. These studies suggested that the mechanism of cross-resistance was underpinned by the increased resilience of the outer membrane of CHX-resistant strains [97,98]. 

Another study reported a marginal increase in susceptibility to CHX, ceftazidime, gentamicin, and chloramphenicol in clinical isolates of *E. coli* adapted to triclosan [72]. However, in the same study, triclosan-resistant strains of *K. pneumoniae* showed reduced susceptibility to CHX [72]. In keeping with this finding, others showed that CHX-adapted strains of *K. pneumoniae* are more resistant to triclosan and other antimicrobial agents, presumably as a result of the upregulation of *acrAB* and *ramA* in turn activating the AcrAB-TolC efflux pump. In this study, the inactivation of AcrAB-TolC resulted in *K. pneumoniae* increased susceptibility to several antibiotics and biocides, including CHX, triclosan, and benzalkonium chloride [74] (Figure 1). In clinical isolates of *Staphylococcus aureus*, overexpression of MDR efflux pump genes *mepA*, *mdeA*, *norA*, and *norC* on exposure to low concentrations of different biocides resulted in an increased resistance to a range of biocides and antibiotics, including CHX [99].

In summary, the main mechanisms thought to underpin cross-resistance include a loss or decrease in OMP, a change in LPS profile and electrostatic activity of the outer membrane, and the activation and/or overexpression of multidrug efflux pumps. MDR efflux pumps may be important mediators of cross-resistance because of the wide breadth of substrate specificity to antimicrobial agents. The chromosomal genes encoding MDR efflux pumps are *lmrS*, *norC*, *norA* (*emeA* in *Enterococci*), *sdrM*, *sepA*, *mdeA*, and *mepA*. However, many MDR genes are carried on plasmids, for example *qacA/B*, *qacG*, *qacH*, *qacJ*, and *smr (qacC*) identified in *Staphylococci* [69,70,99,100,101], thus representing a concern for intra-species and inter-species spread through horizontal gene transfer. Selective environmental pressure by the intensive use of CHX may also drive the overexpression of MDR efflux pumps genes. Table 4 shows the mechanisms and genes implicated in cross-resistance between CHX and other microbial agents.

It is important to emphasise that all reports of cross-resistance between CHX and other antimicrobials identified in this review are limited to reduced susceptibility to CHX generated in vitro through bacterial exposure to gradually increasing sub-bactericidal concentrations of CHX. This experimental approach draws attention to the potential importance of cumulative exposure to CHX over time in the increased emergence and spread of antimicrobial resistance. However, observations of in vitro-generated mutants do not take into account the role of bacterial fitness. As such, the findings of these studies may not hold true when analysing fresh environmental or clinical isolates. Clearly, speculations regarding cross-resistance between CHX and other antimicrobial drugs will require confirmation in aetiological and interventional clinical studies.

## 9. CHX Resistance in Intensive Care Units

The main purpose of CHX use in intensive care settings is the prevention of bacteremia through skin and mucous membrane decontamination as well as disinfection of inanimate surfaces and medical equipment. In this clinical setting, antimicrobial resistance represents a major challenge due to intensive antibiotic prescribing and use of topical antimicrobials.

Large population-based studies concluded that CHX bathing in intensive care units is still an effective measure for the reduction of healthcare-associated infections [103,104,105], notwithstanding some studies showing no statistically significant reductions in infections [106,107,108]. Over the years, concerns have been raised regarding the potential of intensive chlorhexidine bathing to cause the increased emergence and spread of antimicrobial resistance. Over a six-year period, the majority of coagulase-negative staphylococcus-related bloodstream infections in an intensive care unit in Scotland, where universal CHX bathing took place, were caused by a multidrug-resistant sequence type of *S. epidermidis* (ST2) which carried *qacA* and displayed reduced susceptibility to CHX. However, no change in CHX susceptibility was reported in *S. aureus.* [67]. A genomic analysis of a global collection of *S. epidermidis* showed that the majority of qacA/B-positive isolates belonged to the multidrug-resistant clone ST2. These findings emphasize the high prevalence of reduced susceptibility to CHX in this *S. epidermidis* lineage and the possibility that exposure to CHX may play a role in the selection of multidrug-resistant clones [109,110].

## 10. CHX Resistance and Oral Biofilm

The effect of CHX on oral microbial biofilms deserves special mention given the widespread use of CHX in oral healthcare. Tooth surfaces, due to their non-desquamating nature, are niches for the sturdiest of microbial biofilms, which allow bacteria to survive against most external challenges. This is particularly true of bacteria populating the deep strata of dental plaque, which are less accessible to antimicrobials; they can adapt to low concentrations of biocides reaching this layer in addition to adapting to low levels of nutrients through reduced metabolic activity [111]. These conditions may facilitate the development of resistance against CHX and other antimicrobial agents, as the microbes are exposed to sub-inhibitory concentrations [62]. Moreover, as mentioned earlier, extracellular DNA embedded in the microbial biofilm may reduce CHX susceptibility by non-specific binding to positively charged CHX [61]. In these conditions, and given the intensive use of CHX in oral healthcare, it is reasonable to speculate that the oral cavity is fertile ground for adaptation and development of resistance to CHX in microbial communities [112], but this has not been formally evaluated as yet.

## 11. Discussion

CHX activity, as that of all biocides, is naturally affected by environmental-related factors, such as pH, temperature, presence of organic matter, concentration gradient, and exposure time, which can be challenging to control in experimental settings [113]. Therefore, methods used for antibiotic susceptibility testing can lead to inappropriate conclusions if applied to biocides. Indeed, the use of subclinical concentrations of CHX in antimicrobial susceptibility testing is a recognised challenge in the study of CHX resistance in vitro.

An important systematic review analysed CHX resistance trends over time in a range of bacteria as well as *C. albicans* [5]. We note that this systematic review was undertaken by one author, thereby raising the possibility of selection bias and analytical bias [114]. Nonetheless, the review suggested a decrease in CHX susceptibility in most of the assessed Gram −ve bacteria. The same review showed a significant decrease over time in CHX susceptibility among *P. aeruginosa* during a period of 80 years, and that clinical isolates of *P. aeruginosa* displayed lower CHX susceptibility than non-clinical isolates. CHX susceptibility over decades decreased marginally in other species. However, this review, as did previous reports [115], highlighted the concern that in vitro CHX-adapted species cannot recapitulate clinical conditions, not least because of the major differential between CHX concentrations used in vitro versus in vivo. The term “resistance” should be used carefully in the study of the antimicrobial activity of biocides. This term has been previously defined as the failure to kill or inhibit growth of a microorganism by a concentration of antimicrobial agent that can kill or inhibit the growth of other strains of the same species in vivo [111]. In the case of CHX susceptibility testing, in vitro assays are typically conducted at concentrations which are several folds lower than those used in clinical practice [5]. Therefore, reduced susceptibility to CHX of any microbial isolates in vitro may not reflect CHX resistance in vivo. Further, interpretation and synthesis of the findings of the plethora of studies examining susceptibility to biocides is complicated by the inconsistent compliance with standard laboratory procedures for biocide susceptibility testing [116].

Importantly, most studies have evaluated clinical isolates, with only a few exceptions evaluating clinical isolates in comparison with environmental isolates. In response to the One Health commitment to tackling antimicrobial resistance [117], future clinical studies should augment the effort to include analysis of environmental isolates in order to advance our understanding of the sources and selective pressure which cause the emergence and spread of resistance to antibiotics and biocides.

With regards to mechanistic studies of CHX resistance and cross-resistance between biocides and antibiotics, most studies focused on the direct effect of MDR efflux pump genes rather than the combined role of multiple genes and other mechanisms implicated in the reduced susceptibility to CHX. In this review, we highlighted the important role of outer membrane proteins and LPS profile changes which can alter the outer membrane charge, in turn altering the susceptibility to a range of antimicrobials [59,77].

## 12. Conclusions Regarding Potential Implications for Clinical Practice

The potential role of CHX use in oral healthcare on the selection pressure driving the increase in antimicrobial resistance should be carefully considered. We recommend raising awareness amongst dental practitioners to motivate patients regarding the importance of professional and mechanical plaque removal prior to the use of CHX. Consideration should be given to regulating the sale of CHX-based oral hygiene products.

CHX is critical for a wide range of clinical uses, which include biological and inanimate surface disinfection, impregnation of catheter and wound dressing, and oral healthcare products. In the context of the concerns highlighted in this review, it would be prudent to educate healthcare professionals regarding the possibility of CHX cross-resistance with antibiotics. Notwithstanding that definitive evidence based on clinical studies is still lacking, the implications of cross-resistance and selection of multidrug resistance by the intensive use of CHX in community and hospital settings could be major.

## Figures and Tables

**Table 1:** Examples of major efflux pump families in Gram-negative and Gram-positive bacteria and their relevance to biocides.

Efflux Pump Family	Type of Energy Needed	Efflux Pumps in Gram +ve (Location of Gene Encoding the Efflux Pump)	Efflux Pumps in Gram −ve (Location of Gene Encoding the Efflux Pump)	Biocide Substrate [Ref.]
ATP-binding cassette family (ABC)	Primary active transporter	EmrA (Chromosome)	MacAB-TolC (Chromosome)	Silver [36]
Small multidrug resistance (SMR) family	Secondary active transporter	QacC (Plasmid)		Quaternary ammonium compounds [37,38,39]
Multidrug and toxin extrusion (MATE) family	Secondary active transporter	NorM (Chromosome)	PmpM (Plasmid)	Benzalkonium chloride [44]
Major facilitator superfamily (MFS)	Secondary active transporter	QacA (Plasmid)	EmrAB-TolC (Chromosome)	Benzalkonium chloride, CHX [38,39]
Resistance nodulation cell division family (RND)	Secondary active transporter		AcrAB-TolC (Chromosome)	Triclosan [43]

**Table 4:** Mechanisms and genes implicated in cross-resistance between CHX and other antimicrobial agents.

Mechanism of Cross-Resistance	Genes Implicated in Cross-Resistance	References
Loss or decrease OMP	*ompA*, *ompC* and *ompF*	[102]
Change in LPS profile	*pmrK*	[76,94,95]
Activation of MDR efflux pumps	*efrA/b*, *acrAB*, *ramA*, *mepA*, *mdeA*, *norA*, *norC*, *lmrS*, *sdrM*, *sepA*, *qacA/B*, *qacG*, *qacH*, *qacJ*, and *smr* (*qacC*)	[69,70,74,91,99,100,101]

## Data Availability

Not applicable.
